# The Clinical Utility of the Adolescent and Young Adult Psycho-Oncology Screening Tool (AYA-POST): Perspectives of AYA Cancer Patients and Healthcare Professionals

**DOI:** 10.3389/fpsyg.2022.872830

**Published:** 2022-05-06

**Authors:** Pandora Patterson, Fiona E. J. McDonald, Kimberley R. Allison, Helen Bibby, Michael Osborn, Karen Matthews, Ursula M. Sansom-Daly, Kate Thompson, Meg Plaster, Antoinette Anazodo

**Affiliations:** ^1^Canteen Australia, Sydney, NSW, Australia; ^2^Faculty of Medicine and Health, The University of Sydney, Sydney, NSW, Australia; ^3^South Australia/Northern Territory Youth Cancer Service, Royal Adelaide Hospital, Adelaide, SA, Australia; ^4^Department of Haematology and Oncology, Women’s and Children’s Hospital, Adelaide, SA, Australia; ^5^Adelaide Medical School, University of Adelaide, Adelaide, SA, Australia; ^6^New South Wales/Australian Capital Territory Youth Cancer Service, Sydney, NSW, Australia; ^7^Behavioural Sciences Unit, School of Clinical Medicine, UNSW Medicine and Health, Randwick Clinical Campus, Discipline of Paediatrics and Child Health, University of New South Wales, Sydney, NSW, Australia; ^8^Victoria/Tasmania Youth Cancer Service, Melbourne, VIC, Australia; ^9^Western Australia Youth Cancer Service, Perth, WA, Australia; ^10^Kids Cancer Centre, Sydney Children’s Hospital, Sydney, NSW, Australia; ^11^Nelune Comprehensive Cancer Centre, Prince of Wales Hospital, Sydney, NSW, Australia; ^12^School of Women’s and Children’s Health, University of New South Wales, Sydney, NSW, Australia

**Keywords:** adolescent and young adult, clinical utility, distress, needs assessment, psycho-oncology, psychosocial screening

## Abstract

**Objective:**

Routine psychosocial screening and assessment of people diagnosed with cancer are crucial to the timely detection of distress and provision of tailored supportive care; however, appropriate screening tools have been lacking for adolescents and young adults (AYAs), who have unique needs and experiences. One exception is the recently validated AYA Psycho-Oncology Screening Tool (AYA-POST) for use with young people aged 15–29 years, which comprises a distress thermometer and age-specific needs assessment. This study investigates the clinical utility of this measure, as well as the subsequent service responsiveness within the Australian Youth Cancer Services.

**Method:**

In total, 118 AYAs and 29 healthcare professionals: (HCPs) completed surveys about the clinical utility of the AYA-POST; a subset of 30 AYAs completed a 3-month follow-up survey assessing service responsiveness. Descriptive statistics (frequencies/means) were computed for all items, with chi-square analyses used to explore whether perceived clinical utility varied with AYA age, AYA sex, HCP discipline or HCP length of time using the AYA-POST.

**Results:**

Participants’ responses demonstrate high levels of satisfaction with the tool, evidencing its appropriateness, practicability and acceptability. Moreover, the AYA-POST was reported to facilitate communication about psychosocial needs and prompt referrals, indicating good service responsiveness. Ratings of clinical utility did not differ significantly between AYA and HCP groups.

**Conclusion:**

This study demonstrates that the AYA-POST is an appropriate tool in the psychosocial screening of AYAs with cancer, facilitating the identification of distress and unique concerns in this population and valuable in triaging and tailoring care for young cancer patients.

## Introduction

A cancer diagnosis in adolescence or young adulthood can cause significant psychosocial disruption during an already dynamic developmental stage. Affected adolescents and young adults (AYAs, 12–25 years) are at greater risk of developing mental health conditions (Barnett et al., 2016; Zebrack et al., 2016), experience disruptions to familial, peer and romantic relationships (Warner et al., 2016) and may have their educational and vocational plans interrupted (Fardell et al., 2017). AYAs with cancer typically report higher levels of psychological symptomatology than other age groups (Li and Deng, 2004; Cardoso et al., 2012; Barnett et al., 2016) and the risk of poorer psychosocial outcomes is particularly pronounced for females, AYAs with poorer physical health or late effects, and those experiencing educational/work disruption or financial precarity (Phillips-Salimi and Andrykowski, 2013; Sansom-Daly and Wakefield, 2013; Yanez et al., 2013). Importantly, distress has been linked to lower health-related quality of life (Greup et al., 2018), greater stress (Hodgson et al., 2021), poorer coping and resilience (Xie et al., 2017; Greup et al., 2018; Hodgson et al., 2021) and lower treatment adherence (Robertson et al., 2015). This is particularly concerning as 50–95% of AYAs reportedly experience unmet supportive care needs (Keegan et al., 2012; Xie et al., 2017) which may persist for years beyond the completion of active treatment (Millar et al., 2010) contributing to ongoing distress (Zebrack et al., 2014). It is therefore crucial to identify and address distress and other contributing and compounding psychosocial issues in AYAs with cancer, to minimise negative impacts and facilitate adjustment and wellbeing.

Routine screening and assessment of the psychosocial wellbeing of AYAs with cancer are crucial to the provision of quality, tailored supportive care and guides responsive and efficient service delivery (Palmer et al., 2014; Zebrack et al., 2016; Patterson et al., 2018; Osborn et al., 2019). In particular, psychosocial screening of all patients can help healthcare professionals (HCPs) to identify those experiencing distress and other concerns in a timely and proactive manner, allowing early intervention to address these issues (Butow et al., 2015; Patterson et al., 2018; Riba et al., 2019). However, the implementation of effective screening, assessment and care pathways relies on the availability of robust, validated psychometric measures to detect distress, something which has historically been lacking for AYAs with cancer (Clinton-McHarg et al., 2010; Wakefield et al., 2013). While there has been some examination of the use of adult distress measures with AYA participants (e.g. Chan et al., 2018), until recently no age-specific tools had been validated across the full AYA age range (Wakefield et al., 2013; Patterson et al., 2021a). When selecting appropriate measures of distress for AYAs with cancer, it is crucial that they reflect the unique needs and experiences of the population, in addition to being psychometrically robust and sensitive to change (Clinton-McHarg et al., 2010).

### The AYA Psycho-Oncology Screening Tool

In 2008, the Australian National Service Delivery Framework for AYAs with Cancer identified the development of age-specific psychosocial assessment tools and processes as a key priority for care (Australian Government, Cancer Australia, and CanTeen, 2008), leading to the subsequent development of the AYA Oncology Psychosocial Care Manual (Canteen, 2011) which includes the AYA Psycho-Oncology Screening Tool (AYA-POST; Palmer et al., 2014; Patterson et al., 2021b; see Supplementary Table 1). This validated tool for young people aged 15–29 years comprises the Distress Thermometer (DT; a single-item measure of psychological distress) and the Needs Assessment (NA; asking patients to indicate if they are concerned about commonly reported concerns). The DT is identical to that used with adult cancer patients: it is recommended for use by the (US) National Comprehensive Cancer Network, has been translated into over twenty languages, with extensive validation work evidencing its strong psychometric properties with adults including sensitivity, specificity and predictive value (Carlson et al., 2012; Donovan et al., 2014). Typically, a cut-off score of 4 on the DT has been used to indicate clinically significant levels of distress in adults (Jacobsen et al., 2005; Donovan et al., 2014), while a cut-off of 5 is more appropriate for AYAs (Patterson et al., 2021b). The accompanying NA was adapted from the adult Problem Checklist (PCL) following indications that the latter did not reflect key AYA concerns (Palmer et al., 2014). Young people with cancer and AYA healthcare professionals consulted on the revision of the original checklist which resulted in fifty issues pertinent to this population spanning six domains: practical needs, family, emotions, social issues, physical symptoms and information (Palmer et al., 2014; Patterson et al., 2021b). The tool also includes an option for AYAs to specify additional concerns they experienced which are not covered on the list. The AYA-POST also includes a checklist of 11 items for clinicians to indicate whether they have discussed key issues with the AYA patient (e.g. clinical trials and fertility preservation), and a joint sign-off by the clinician and AYA to confirm they have completed the tool, understand the process and have been informed of next steps (Patterson et al., 2018).

The AYA Oncology Psychosocial Care Manual and AYA-POST are used nationally in Australia by the hospital-based Youth Cancer Services (Patterson et al., 2021a) and have been recommended for use by the Clinical Oncology Society of Australia (Psychosocial Management of AYA Cancer Patients Working Group, 2011), as well as being translated for use internationally. The AYA-POST has recently been validated with an international cohort of AYAs with cancer, where it was found to have good convergent validity, with a DT cut-off score of 5 providing acceptable specificity and sensitivity scores for use as a screening tool, and the NA items being highly relevant to this age group (Patterson et al., 2015, 2021b). While this is important and necessary, it is not sufficient in ensuring a tool is useful for clinical practice; its clinical utility also needs to be examined.

### Clinical Utility

Smart (2006) conceptualises clinical utility as ‘a multidimensional judgement about the usefulness, benefits and drawbacks of an intervention’, identifying four key components: appropriateness, accessibility, practicability and acceptability (Smart, 2006). In brief, appropriateness comprises both evidence of efficacy of an instrument and perceptions of its relevance to a particular population. Accessibility covers both economic and logistical issues around resourcing—the procurement and cost of materials. Practicability assesses the functionality and suitability of the materials, as well as if users have the knowledge or training to use them. Finally, acceptability is assessed from the perspectives of clients, HCPs and broader society. In addition, it is also important to consider service responsiveness. The efficacy of distress screening programmes depends not only on the use of screening to identify patients in need, but also further assessment of psychosocial issues, triaging to appropriate services and evidence-based treatment (Carlson, 2013), which is ‘where the real impact [of screening] is felt’ (Smith et al., 2018). Notably, service responsiveness is context-specific and does not meaningfully generalise beyond the service or programme which is evaluated.

Research into the clinical utility of the DT and PCL in adult populations has thus far focused on its ability to accurately identify patients experiencing clinically significant levels of distress and, to a lesser extent, its acceptability; relatively little work has explored its accessibility, practicability or service responsiveness (Snowden et al., 2011). It is unclear whether use of the DT/PCL improves patient outcomes, perhaps because screening has not consistently led to increased referrals for support in these implementation studies (Snowden et al., 2011). More recent research has largely replicated these findings (e.g. Hollingworth et al., 2013; Williams et al., 2015; Linehan et al., 2017; Van der Meulen et al., 2018), confirming the acceptability of the measures to patients and HCPs but drawing further attention to the need to consider service responsiveness in tandem with more commonly investigated aspects of clinical utility, as the benefits of screening are contingent on its use to provide referrals to appropriate support services and facilitate their uptake.

Since the experience of completing a questionnaire or participating in clinical research may differ based on participant characteristics such as age and gender (Lee et al., 2013; Knäuper et al., 2016), and/or features of the researcher/administrator such as qualifications or expertise (Kost et al., 2011), exploring individual differences relating to these factors, have the potential to provide useful additional information about the generalizability of clinical utility findings.

### Present Study

This study is the first to explore the clinical utility of the AYA-POST and subsequent service responsiveness of the Australian Youth Cancer Services (YCS). Perspectives were sought from both AYAs receiving care within the YCS and the HCPs who work with them. The primary aim is to evaluate the appropriateness, practicability and acceptability of the AYA-POST, as well as the service responsiveness of the YCS; a secondary aim was to explore whether perceptions of clinical utility varied between subgroups of AYAs (by gender or age) and HCPs (by discipline or length of time using the AYA-POST). The data collected in this study are part of a larger study that examined the validity of the AYA-POST and identified predictors of distress and psychosocial concerns (Patterson et al., 2015).

## Materials and Methods

### Setting

In Australia, healthcare is provided by a combination of public and private health systems: citizens and permanent residents are able to access universal healthcare through Medicare, which allows free or subsidised access to medical services, hospital treatment and prescription medications (Australian Government Department of Health, 2020), while private health insurance allows greater choice of practitioners and hospitals, and covers other health services and expenses (e.g. physiotherapy and psychology) (Australian Government, 2019). These systems are supplemented by non-government organisations providing health information, counselling services and peer support, among other services (e.g. Lifeline for crisis support and suicide prevention; state Cancer Councils for cancer information and support). The majority of AYAs with cancer are treated in public hospitals (Osborn et al., 2013). Approximately 75% of those requiring hospital-based care are treated through the specialised Youth Cancer Services (YCS), which provide age-appropriate, holistic cancer care to 15–25 year olds across Australia (CanTeen Australia, 2015, 2017; Patterson et al., 2021a). The YCS comprises five jurisdictions covering all Australian states and territories, which have lead sites in major hospitals and work in collaboration with a network of hospitals, health services and HCPs around the nation, allowing AYAs to benefit from both the age-specialised care offered by the YCS and disease-specific expertise of local cancer teams (Patterson et al., 2021a). A key feature of the YCS is their integrated, multidisciplinary approach to cancer care, with teams comprising medical, nursing, allied health and support professionals, and close ties with community organisations (e.g. Canteen for AYA-specific cancer information and support; Patterson et al., 2021a). The psychosocial care pathway implemented in the YCS includes routine screening, assessment and care planning, as detailed in the AYA Oncology Psychosocial Care Manual (Canteen, 2011), to ensure that the concerns of AYAs are detected and addressed in a timely and systematic way (Patterson et al., 2018, 2021a). The use of the AYA-POST is recommended as part of the screening process. Critically, the interconnected and multidisciplinary nature of the YCS provide a rich environment for YCS patients’ psychosocial needs to be identified and addressed through internal and external referrals, optimising service responsiveness.

### Design

The clinical utility of the DT was evaluated in accordance with Smart’s multidimensional clinical utility framework, incorporating both AYA and HCP perspectives.

AYA data collection involved surveys at two time points. The T1 survey was completed within 3 months of diagnosis and included demographic and cancer details, the AYA-POST and questions on the clinical utility of the measure, adapted from Breen et al.’s (2012) work. This survey also contained measures used in the broader validation study (see Patterson et al., 2015, 2021b for further details). The T2 survey was an optional component of the study, intended to be completed by a subset of T1 participants during a follow-up phone interview approximately 2 months later. This survey included the re-administration of the AYA-POST as well as questions on service responsiveness.

HCP perspectives were collected using an online survey, which included questions about the clinical utility of the AYA-POST and barriers to screening for distress.

The study received ethical approval from the Human Research Ethics Committees at seven lead sites across the country: ACT Health (ETH.11.14.331), Children’s Health Queensland Hospital and Health Service (HREC/14/QRCH/374), Northern Territory Department of Health and Menzies School of Health Research (HREC-2014-2,275), Peter MacCallum Cancer Centre (14/178), Prince of Wales Hospital (HREC/14/POWH/261), Sir Charles Gairdner Hospital (2015–048) and the Women’s and Children’s Hospital (HREC/14/WCHN/113).

### Participants and Recruitment

AYA participants were recruited through the five state/territory YCS, where a nominated team member was responsible for identifying eligible AYAs and providing participant information and consent forms. AYAs were eligible to participate in the broader AYA-POST validation study if they were aged between 15 and 25 years, had been diagnosed with any cancer in the preceding 3 months, were receiving treatment (any type) at a YCS-affiliated hospital and were assessed by the recruiting clinician as able to complete the survey (e.g. adequate English proficiency). Eligible young people were invited to the study by a research assistant/nurse at their hospital, who provided them with an invitation letter from the research team and a participant information and consent pack. Consenting young people completed paper versions of the questionnaire pack and indicated whether they were interested to take part in an optional T2 interview a few months after completing the T1 surveys. T2 interviews were conducted by members of the research team over the telephone.

HCPs were eligible to participate if they were employed by the YCS during the data collection period. They were invited to complete the online survey by email.

### Measures

#### AYA Clinical Utility Survey

After completing the AYA-POST at T1, AYAs completed several closed and open-ended questions assessing the tool’s clinical utility. These items were adapted from Breen et al.’s (2012) work (see Patterson et al., 2015, for details). This included eight items on the appropriateness, practicability and acceptability of the tool (Table 1), which participants responded to using a five-point rating scale (1 = ‘strongly agree’ and 5 = ‘strongly disagree’). AYAs were also asked if the tool covered the main areas they needed (yes/no), and if there were any other questions that should be asked (open-ended).

**Table 1:** Examples of items assessing appropriateness, practicability, acceptability and service responsiveness of the AYA-POST.

Construct	Example Items
Appropriateness	The tool covered issues that were relevant to me (AYA T1 survey)
	The tool covered issues I thought were important for AYA cancer patients (HCP survey)
Practicability	The language in the tool was easy to understand (AYA T1 survey)
	Administering the tool has slowed down or interfered with clinical operations (HCP survey)
Acceptability	I would be happy to complete the tool again as part of my future care (AYA T1 survey)
	I would be happy to administer the tool to future patients (HCP survey)
Service responsiveness	After completing the tool last time, my medical care team made me aware that help was available if I needed it (AYA T2 survey)
	The tool helped patients receive appropriate follow-up (HCP survey)

#### AYA Service Responsiveness Survey

During the T2 interview, participants responded to seven items about how completing the AYA-POST at T1 may have impacted the care they received from HCPs (Table 1) using the same five-point rating scale (1 = ‘strongly agree’ and 5 = ‘strongly disagree’). Three of these items were adapted from Breen et al.’s (2012) work; four additional items were developed to assess whether the AYA-POST facilitated the provision of useful information and referrals, and increased comfort in discussing and seeking help for emotional and psychological needs. Participants were also asked how many referrals they had received for their needs (open-ended).

#### HCP Clinical Utility Survey

HCPs completed an online survey assessing the utility of the AYA-POST. Two open-ended questions asked participants if there were topics not covered in the AYA-POST that were relevant to AYAs, and if there were topics covered in too much detail. HCPs then indicated the extent to which 10 factors (e.g. ‘lack of time’) were barriers to screening for distress, using a five-point Likert scale from 1 (‘not at all a barrier’) to 5 (‘very much a barrier’). Finally, HCPs who had previously used the AYA-POST rated their agreement with eleven items assessing the appropriateness, practicability and acceptability of the tool (Table 1) using a five-point Likert scale from 1 (‘strongly agree’) to 5 (‘strongly disagree’). These items were adapted from previous work on the acceptability of and barriers to distress screening among HCPs (Tavernier et al., 2013; Ristevski et al., 2015; see Patterson et al., 2015, for details).

### Data Cleaning and Analysis

Participants who did not complete any clinical utility measures were excluded from analyses. Given the low prevalence of missing data, these responses were not imputed. Responses using the five point *strongly agree*—*strongly disagree* scale were collapsed into three categories (‘strongly agree/agree’, ‘unsure’ and ‘disagree/strongly disagree’) for ease of interpretation. Responses using the five point *not at all a barrier—very much a barrier* scale were similarly collapsed as: 1–2 = ‘not a barrier’, 3 = ‘somewhat a barrier’ and 4–5 = ‘barrier’.

Descriptive statistics (frequencies, means and standard deviations) were computed to assess clinical utility (appropriateness, practicability and acceptability) and service responsiveness. Chi-square analyses were used to explore whether perceived clinical utility differed according to AYA age (15–20 years vs. 21–25 years), AYA sex (female vs. male), HCP discipline (nursing vs. allied health; medical professionals excluded) or HCP length of time using the AYA-POST (<3 years vs. >3 years). A Bonferroni correction was applied to correct for the elevated probability of Type I errors when conducting multiple comparisons. The corrected cut-off for significance was *p* = 0.006 for the eight AYA analyses, and *p* = 0.002 for the 21 HCP analyses.

As open-ended responses to questions about items which could be added/removed were few and brief, formal qualitative analysis was not considered appropriate. Instead, commonalities were identified and grouped in order to summarise participant suggestions for item inclusion/deletion.

## Results

### Participant Characteristics

In total, 118 AYAs (15–25 years, *M* = 20.7 years, SD = 3.2 years; 57 females, 61 male) completed the T1 survey either alone (43.2%) or with family/a partner (27.1%), a HCP (31.4%) and/or another patient (0.8%). Thirty (*M* = 22.1 years, SD = 2.3 years; 17 females, 13 male) chose to complete the T2 interview approximately 3 months later (*M* = 86.9 days, SD = 50.4; range 48–274). Twenty-nine HCPs (medical, nursing and allied health) completed the HCP survey. Table 2 provides further demographic information about these participants, as well as analyses comparing T2 respondents and non-respondents (T2 respondents were slightly older than non-respondents but did not significantly differ in any other respect).

**Table 2:** Demographics of participating AYAs and HCPs.

Demographic	*n* (%)		Test of difference^**^	
Adolescents and young adults (T1)	T1 participants (*N* = 118)	T2 participants (*N* = 30)	*χ* ^2^	*p*
Sex			1.126	0.289
Female	57 (48)	17 (57)		
Male	61 (52)	13 (43)		
Age			4.893	0.027
15–20 years	55 (49)	7 (24)		
21–25 years	57 (51)	22 (76)		
Cultural and linguistic background^*^				
Aboriginal and/or Torres Strait Islander	5 (4)	2 (7)	0.585	0.444
Born overseas	15 (13)	2 (7)	1.367	0.242
Speaks another language at home	16 (14)	3 (10)	4.149	0.126
Location			0.209	0.901
Metropolitan	84 (71)	21 (70)		
Rural	28 (24)	7 (24)		
Remote	6 (5)	2 (7)		
Employment (at diagnosis)^*^			–	–
Working	68 (58)	19 (63)		
Studying	58 (49)	12 (40)		
Home duties	5 (4)	2 (7)		
Volunteering	2 (2)	1 (3)		
On leave	1 (1)	1 (3)		
Unemployed, looking for work	11 (9)	2 (7)		
Cancer types^*^			–	–
Lymphoma	40 (34)	13 (43)		
Leukaemia	23 (19)	4 (13)		
Sarcoma	21 (18)	3 (10)		
Testicular	15 (13)	5 (17)		
Brain/central nervous system	8 (7)	2 (7)		
Ovarian	3 (3)	0		
Breast	2 (2)	1 (3)		
Colorectal	2 (2)	1 (3)		
Other	13 (11)	2 (7)		
Treatment status			2.014	0.365
Not yet started	12 (10)	5 (17)		
On treatment	103 (87)	24 (80)		
Not sure	3 (3)	1 (3)		
	*M* (SD), range	*M* (SD), range	*F*	*p*
Age at survey completion (years)	20.7 (3.2), 15–25	22.1 (2.3), 16–25	7.594	0.007
Age at diagnosis (years)	20.1 (3.2), 14–25	21.5 (2.3), 16–25	8.996	0.003
Healthcare professionals	Participants (*n* = 29)			
Discipline				
Medical	3 (10)			
Nursing	15 (52)			
Psychology	4 (14)			
Social work	5 (17)			
Youth work	2 (7)			
Received training on AYA psychosocial distress screening				
At YCS workshop	14 (47)			
On the job	10 (36)			
	*M* (SD), range			
Time in role (months)	41.7 (41.8), 2–128			

* AYAs could report multiple of these options, if applicable.

** Between T2 respondents and non-respondents.

### AYA Perspectives on Clinical Utility

**Figure 1:**
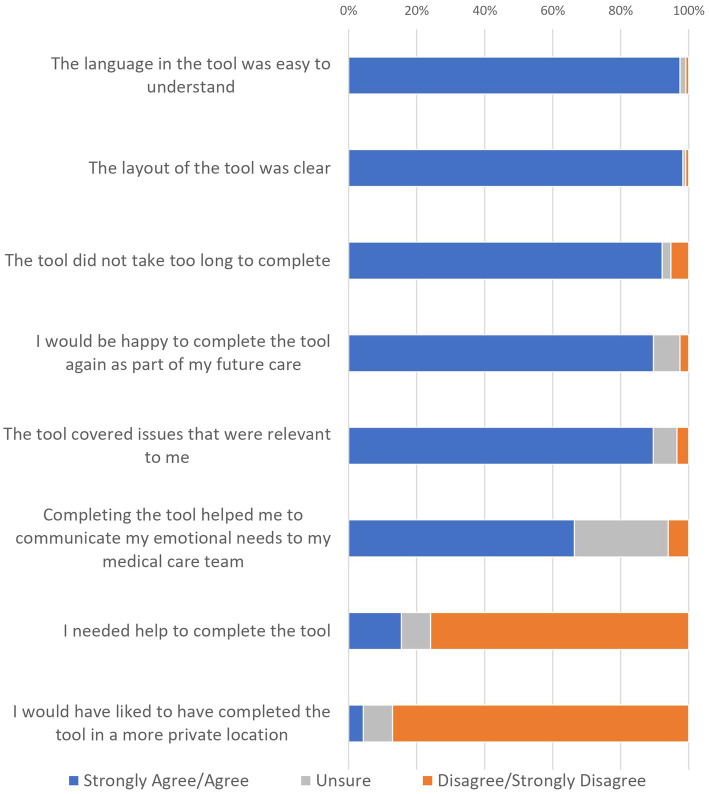
AYA ratings of the acceptability of the AYA-POST.

Immediately after completing the AYA-POST at T1, AYAs generally agreed that the tool was acceptable: clear (98%) and easy to understand (97%), relevant (90%) and helpful in communicating emotional needs to their healthcare team (66%). They also reported not needing help to complete the tool (76%). Chi-square analyses indicated there were no evidence of significant differences in ratings by AYA age or sex. Figure 1 shows the response to all AYA acceptability questions.

Almost all (95%) of AYAs agreed that the AYA-POST had covered all the main areas of their needs. Eight participants reported that they had experienced concerns not included in the NA related to treatment (e.g. delays), social activities (e.g. missing specific hobbies), physical effects (e.g. tinnitus), emotions (e.g. homesickness) or information needs (e.g. next steps for follow-up).

Similarly, some participants’ suggestions of additional items which could be included were already included in the NA (e.g. educational and employment concerns) or overlapped with existing items (e.g. sport may be covered by ‘missing doing the “normal stuff” with friends’). Participants also suggested heath and healthcare concerns which were not entirely captured by the ‘other medical worry’ category—for example, relationships with the medical team, concerns about slow healthcare systems.

### AYA Reports of Service Responsiveness

**Figure 2:**
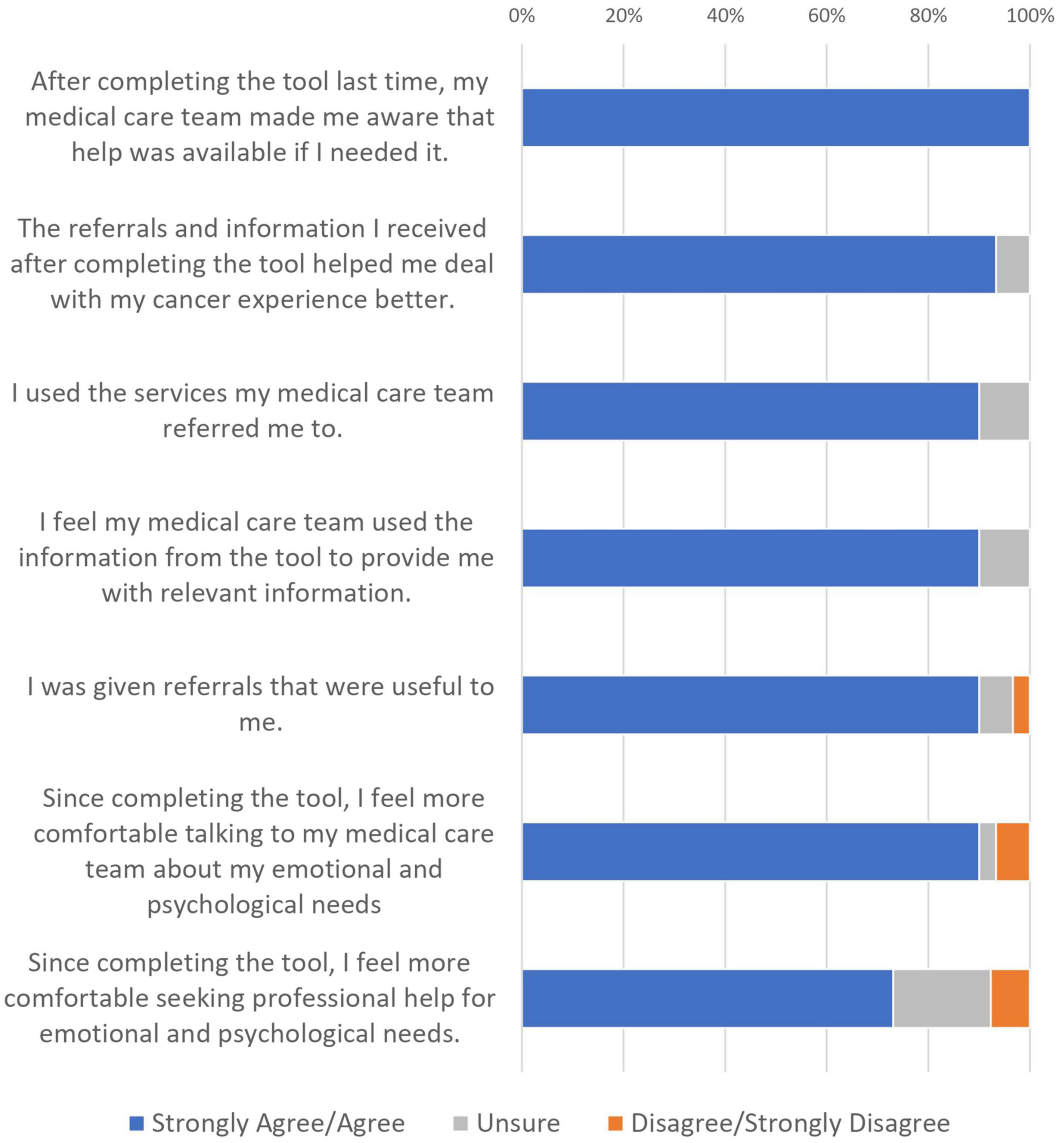
AYA ratings of service responsiveness since completing the AYA-POST.

At T2, participating AYAs generally agreed that the care they had received had improved since completing the AYA-POST (Figure 2): for example, they reported being given useful information (90%) and referrals (90%). Almost all (90%) of these participants had used the services they were referred to, and these reportedly helped them to adjust to their cancer experience (93%). They also reported being more comfortable discussing (90%) and seeking help (73%) for their emotional and psychological needs since completing the AYA-POST. When asked how many referrals they had received since T1, participants reported 2.83 referrals on average (range 0–10, SD = 2.60), with 87% of respondents having received at least one referral.

### HCP Perspectives on Clinical Utility

**Figure 3:**
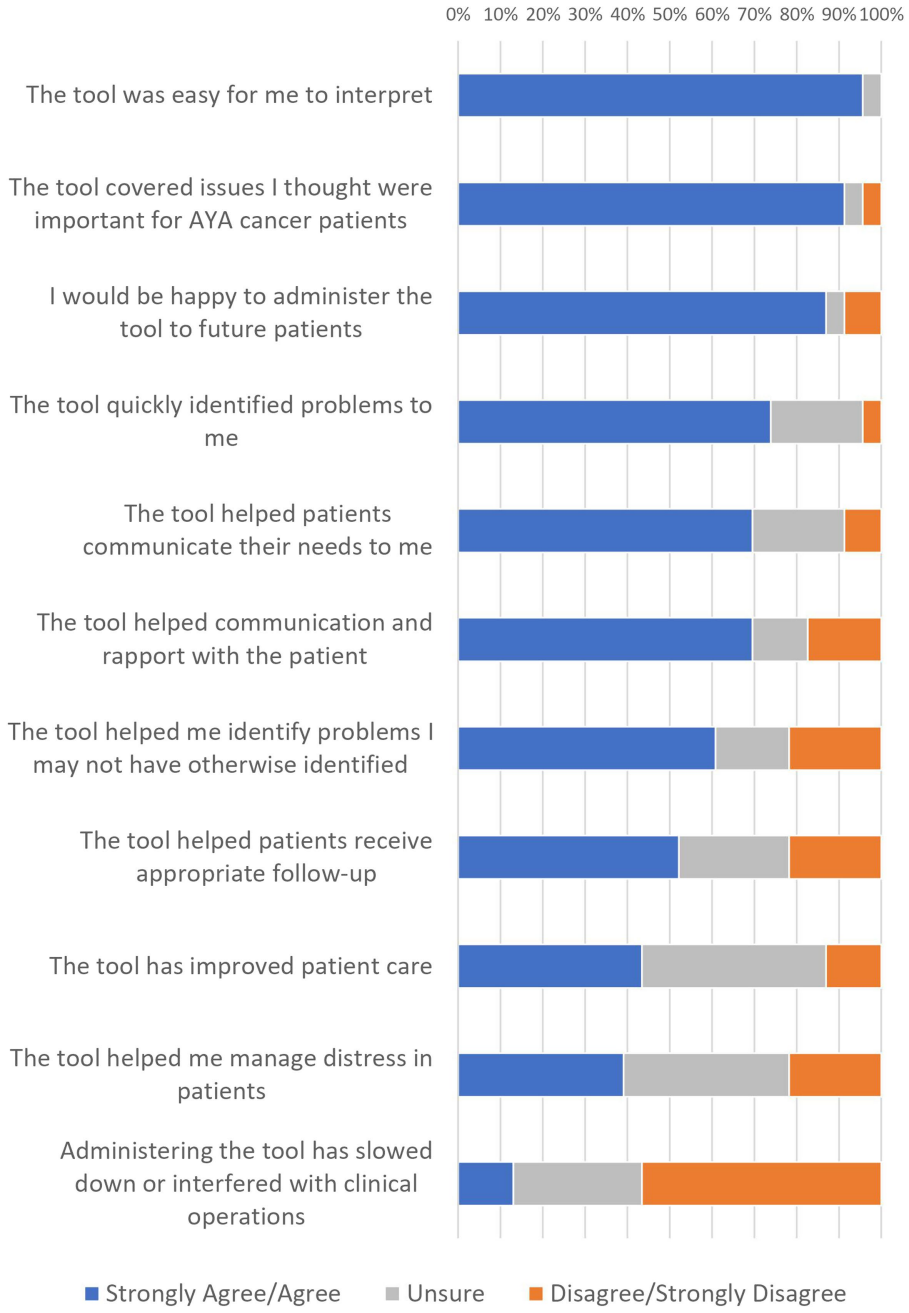
HCP ratings of the clinical utility of the AYA-POST.

Of the 29 HCPs, 23 reported having used the AYA-POST in their clinical practice; almost half (48%) had been using the tool for over 3 years. HCP ratings of the clinical utility of the AYA-POST (for the 23 who reported previous use) are displayed in Figure 3. While HCPs agreed overall that the AYA-POST was acceptable, relevant and feasible, approximately 40% felt it helped them manage patient distress or improve patient care. Chi-square analyses indicated that the distribution of responses did not differ significantly by HCP discipline (nursing vs. psychosocial) or time using the AYA-POST (<3 years vs. >3 years). Of note, reflecting the perceived feasibility of the tool, HCPs mostly did not perceive that administering the tool slowed down or interfered with clinical processes.

Six HCPs suggested additional items which could be added to the AYA-POST; these included difficulties obtaining financial support from the government, menstrual disruption and social media/technology concerns. No HCPs identified items that were irrelevant or could be removed from the NA.

### HCP Reported Barriers to Screening for Distress

**Figure 4:**
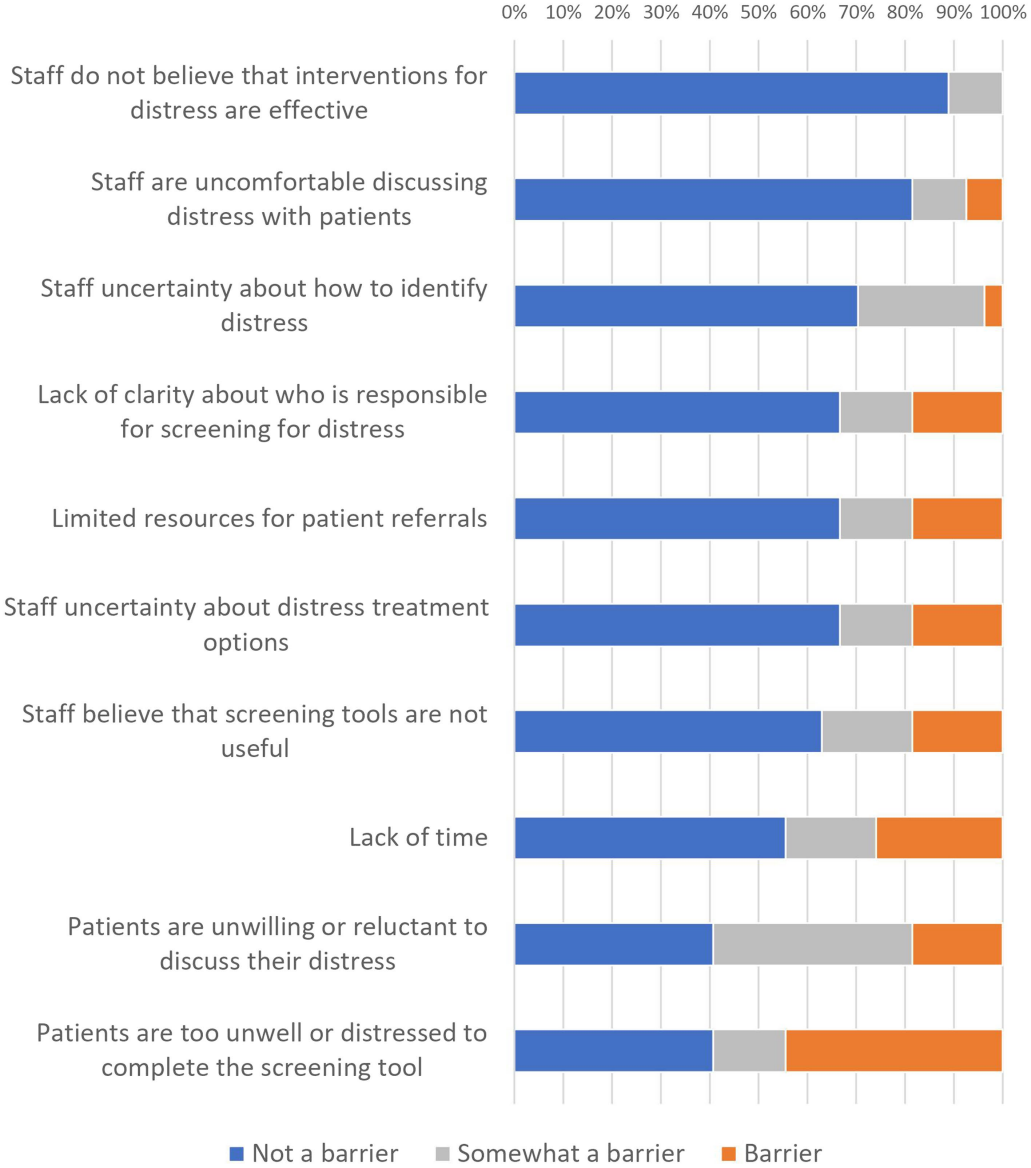
HCP ratings of barriers to use of the AYA-POST.

Overall, results indicated HCPs reported few perceived barriers to screening, with the most common being patients were too unwell or distressed (44% barrier and 15% somewhat), and patients being unwilling or reluctant to discuss distress (19% barrier and 41% somewhat). Figure 4 shows the proportion of HCPs who indicated that each item was a barrier. Chi-square analyses found no evidence that the distribution of responses differed significantly by HCP discipline (nursing vs. psychosocial) or time using the AYA-POST (<3 years vs. >3 years).

## Discussion

The findings of this study demonstrate the clinical utility of the AYA-POST, with both AYAs and HCPs rating the tool as broadly appropriate, practicable and acceptable. Additionally, the tool reportedly facilitated communication about emotional and psychosocial needs, and its use prompted referrals which were overwhelmingly experienced as helpful, indicating good service responsiveness. Results also indicated HCPs reported few perceived barriers to screening, with the most common being patients were too unwell or distressed. Together with findings from the international validation study (Patterson et al., 2021b), this study provides strong support for the AYA-POST as a suitable measure for use as standard clinical practice in the biopsychosocial screening of AYAs with cancer, helping to identify distress and unmet needs among patients and facilitating the triaging and tailoring of care. This is a particularly important development, given the absence of validated, population-specific psychosocial measures for this group (Clinton-McHarg et al., 2010; Palmer et al., 2014).

While HCPs agreed overall that the AYA-POST was acceptable, relevant and feasible, around 40% felt the tool helped them manage patient distress or improve patient care and about the same percentage were unsure. Interestingly, these two items had the highest ‘unsure’ ratings by HCPs when assessing elements of clinical utility. In the absence of further information, it is difficult to know why these two items presented the greatest uncertainty for HCPs; importantly, when looking at AYA patients’ reported experiences of care these concerns were not evidenced, indeed there was substantial reporting on the benefit of the tool in improving their care and helping them manage their emotions. Further research may be useful to better understand the HCP’s responses.

Smart’s model of clinical utility defines appropriateness in terms of the efficacy and perceived relevance of a tool (Smart, 2006). While the concurrent validation study confirmed the ability of the AYA-POST to identify patients experiencing clinically significant levels of distress with acceptable sensitivity and specificity using a cut-off score of 5 (Patterson et al., 2021b), this study indicated that both AYAs and HCPs perceived the measure to be effective in terms of facilitating communication about psychosocial distress and needs. Both groups largely agreed that the AYA-POST helped patients to communicate about their needs with HCPs. AYAs additionally noted that it made them more comfortable to talk about their emotional and psychological needs and seek professional help, while HCPs reported that the tool helped with communication and rapport building. While the efficacy of distress screening measures and processes is typically assessed in terms of their performance at identifying and ameliorating psychosocial issues (e.g. Carlson et al., 2012), this indicates a secondary benefit of screening using the AYA-POST in introducing and legitimising discussion of psychosocial issues. This has also been suggested in previous studies of the DT with adults (Dabrowski et al., 2007; Thewes et al., 2009; Johnson et al., 2010) and has the potential to improve engagement with subsequent psychosocial assessment and treatment.

This study also provided evidence of the relevance of the tool to AYAs diagnosed with cancer. For example, almost all AYAs and HCPs agreed that the AYA-POST covered issues thought to be important for this population, and likewise nearly all AYAs reported that the tool covered their main areas of need. Few participants nominated unique concerns which they were experiencing or thought should be included which were not covered to some extent by the existing items, and no AYA or HCP nominated items for removal. That few recommendations for improvement were made indicates that the NA successfully captures the full range of AYA-specific concerns, which provides support for the content validity of the tool (Haynes et al., 1995). This is consistent with findings from the international validation of the AYA-POST, which reported that five of the 10 most commonly nominated issues were AYA-specific additions not present in the adult PCL (Patterson et al., 2021b). Both AYAs with cancer and HCPs specialising in AYA oncology were involved in the development of the AYA-POST (Palmer et al., 2014), and the positive findings around the tool’s appropriateness (acceptability and practicability) are a testament to the success of this participatory design approach in ensuring stakeholders’ perspectives inform research and service delivery. This is key in ensuring that a psychosocial screening measure is effective.

The practicability of a measure captures the functionality and suitability of materials, as well as whether users have sufficient knowledge and training to use it (Smart, 2006). The surveyed AYAs almost universally agreed that the language and layout used in the AYA-POST were clear, while only a small proportion reported needing help to complete the tool. HCPs were similarly positive about the practicability of the AYA-POST: the majority of those surveyed agreed that it was easy to interpret and quickly identified problems to them, including problems which they may not otherwise have identified. Likewise, AYAs broadly agreed that the measure did not take too long to complete and few HCPs felt its administration slowed down clinical operations; this echoes previous research which has found that the introduction of standardised psychosocial assessment does not increase consultation times (Engelen et al., 2012) and that clinicians are largely satisfied with the time needed to complete these measures (Teela et al., 2020). These considerations are crucial in selecting an instrument to screen for distress, ensuring that both patients and HCPs can easily understand the measure, minimising the need for further explanation or training. Interestingly, HCP ratings of practicability did not differ between nursing and psychosocial staff; this may be due to the strong holistic focus of the YCS, established protocols around psychosocial care and high uptake of training on distress screening among participants (83%). The AYA Oncology Psychosocial Care Manual offers more detailed recommendations as to how the AYA-POST may be interpreted and implemented as part of a more comprehensive assessment and care pathway (Canteen, 2011), and this may be useful in supporting HCPs with less psychosocial training to use the tool in their work.

The acceptability of the AYA-POST to patients and HCPs is largely reflected in their accounts of the measure’s clarity, relevance and helpfulness discussed above; additionally, 90% of AYAs and 87% of HCPs reported that they would be happy to complete or administer the AYA-POST again. These ratings did not differ significantly between female and male AYAs, or between younger and older AYAs; further work may be useful in exploring whether the measure is similarly acceptable to groups underrepresented in this study (and research more broadly) who may have different needs and experiences of care, such as AYAs from culturally/linguistically diverse backgrounds, who are LGBTQI+, or who have disabilities (Wakefield et al., 2013). Of note, this study did not explicitly explore the accessibility of the AYA-POST.

As Carlson (2013) notes, the success of a distress screening process depends not only on the properties of the tool itself, but also on how the health service responds to the results of screening. Previous evaluations of distress screening programmes have shown that screening does not always translate into referral and uptake of psychosocial support services (Carlson, 2013; Mitchell, 2013; Funk et al., 2016), and this lack of follow-up may underlie the limited benefits evidenced for some screening programmes (Carlson, 2013; Mitchell, 2013). By comparison, results from the follow-up AYA survey indicated that the AYA-POST helped to facilitate the provision of appropriate information and referrals to meet patients’ needs. The majority of AYAs reported that since completing the tool, their HCPs had made them aware of help available to them and provided relevant information and useful referrals. Uptake of these referrals was reportedly high, more so than previously reported for AYAs [78% (Ellis et al., 2009)] and older adults [30–40% (Ellis et al., 2009; Johnson et al., 2017)]. AYA participants further indicated that these services helped them to better deal with their cancer experience. These results are encouraging and importantly emphasise the associations of targeted referrals and efficient and effective early intervention with administering the AYA-POST. Highlighting these outcomes in training on the tool and institutionalising its use will increase uptake and maximise the benefits it provides for AYAs. It is also worthwhile noting that screening using the AYA-POST may have greater psychosocial benefit for AYA patients being treated within a model of care such as the YCS due to the multidisciplinary nature of YCS teams, together with their strong ties to youth-based community organisations, providing a rich network of internal and external appropriate supports to whom AYAs can be referred to and in a timely manner (Osborn et al., 2019; Patterson et al., 2021a).

Interestingly, the surveyed HCPs were more reserved in their assessment of whether the AYA-POST impacted care: just over half reported that the tool had helped patients receive appropriate follow-up (52%), while 39 and 43% indicated that its use had helped them manage distress and improve patient care, respectively. This discrepancy suggests that HCPs may underestimate the benefits of using a screener like the AYA-POST; being informed of the current results from AYA patients on the usefulness of the tool and subsequent referrals could help HCPs to better understand this.

Finally, overall results indicated HCPs reported few perceived barriers to screening, with the most common being patients were too unwell or distressed (44% barrier and 15% somewhat), and patients being unwilling or reluctant to discuss distress (19% barrier and 41% somewhat). HCP concerns about AYAs’ illness, distress and reluctance were not evidenced in the responses of AYAs surveyed here. However, this may be influenced by sampling biases; AYAs’ decision to participate in a study on distress screening may be an indicator of their openness to discussing psychosocial issues. Certainly, AYA reluctance to discuss distress has been previously identified as a barrier to accessing psychosocial support, which has been attributed to personal preferences for internalised coping (Holland et al., 2020). It may be that a concise, needs-based measure like the AYA-POST offers a more palatable route to discussing distress for patients who may otherwise be reluctant to engage in interview style assessments. Further research is needed to determine this. System-level barriers around resourcing and responsibility are more frequently reported in the literature (Fradgley et al., 2019; Knies et al., 2019) and suggest a need for services to increase investment in psychosocial staffing to ensure that all AYAs have access to quality care, particularly where patient numbers are expected to increase.

While this study was conducted in the context of the Australian YCS, which is notable for its strong emphasis on age-appropriate, holistic and multidisciplinary cancer care (Osborn et al., 2013; Patterson et al., 2021a), the positive benefits and practice implications discussed throughout have the potential to be similarly realised within international operating environments. It is possible that the positive HCP ratings of the clinical utility of the AYA-POST may to some extent reflect the context of the YCS which places significant emphasis on the psychosocial needs of young people with cancer and has established protocols around the provision of supportive care, and these views may not generalise to HCPs working in non-AYA-specific services or those placing less emphasis on psychosocial care. By contrast, we would expect AYAs’ positive views on the AYA-POST to be more broadly generalisable across settings, although we encourage further research exploring its relevance and appropriateness with young people from underrepresented groups who may have different needs and/or experiences of care. We acknowledge however that AYAs who were more ill or distressed, or who were less open to discussing psychosocial concerns, may be underrepresented among participants (particularly among the small number who chose to complete the T2 survey). Indeed, anecdotal accounts from YCS HCPs involved in recruiting AYAs for this study indicated that some HCPs elected not to promote this project to patients they considered too unwell or distressed, meaning that the clinical utility of the AYA-POST among this subgroup of AYAs is less certain. However, as clinician burden made the collection of data on response rates and non-respondents unfeasible, it was not possible to confirm this.

## Conclusion

Overall, this work indicates high AYA and HCP satisfaction with the AYA-POST, demonstrating its acceptability, practicability and appropriateness in ensuring AYAs with cancer receive appropriate psychosocial care. Combined with concurrent work validating the tool with an international cohort of young people (Patterson et al., 2021b), the study provides strong evidence to support the use of the AYA-POST in psychosocial screening and care provision for these patients, as well as demonstrating the feasibility of using the measure to provide tailored care and referrals. Use of the tool assists in standardising universal screening and referral processes, improving consistency of care (Kim et al., 2018). It can also be useful in detecting psychosocial concerns among the broader AYA cancer population who may not consider themselves to be at risk or who are hesitant to express concerns and support needs themselves, and in detecting issues in domains which may be overlooked, avoided or mistakenly assumed to not be a concern/relevant in non-standardised assessment processes (Skaczkowski et al., 2018).

The AYA-POST can also serve as a useful strategic service planning tool. Identifying commonly reported areas of concern, the data gleaned from its administration can be used to better understand the psychosocial experience of young people with cancer, ensure (or advocate for) sufficient hospital-based services and/or the establishment of clear referral pathways to community-based support to address these issues. Policy makers and health ministries alike can also utilise AYA-POST information in their consideration of developing evidence-based patient-focused models of care for young people with cancer.

## Data Availability Statement

The original contributions presented in the study are included in the article/Supplementary Material, further inquiries can be directed to the corresponding author.

## Ethics Statement

The studies involving human participants were reviewed and approved by the ethical approval from the Human Research Ethics Committees at seven lead sites across the country: ACT Health (ETH.11.14.331), Children’s Health Queensland Hospital and Health Service (HREC/14/QRCH/374), Northern Territory Department of Health and Menzies School of Health Research (HREC-2014-2,275), Peter MacCallum Cancer Centre (14/178), Prince of Wales Hospital (HREC/14/POWH/261), Sir Charles Gairdner Hospital (2015–048) and the Women’s and Children’s Hospital (HREC/14/WCHN/113). Written informed consent from the participants’ legal guardian/next of kin was not required to participate in this study in accordance with the national legislation and the institutional requirements.

## Author Contributions

PP and FM: conceptualization and project administration. PP, FM, KA, and HB: formal analysis. PP, FM, HB, MO, KM, and AA: investigation. PP, FM, US-D, and AA: methodology. PP and KA: writing—original draft. PP, FM, KA, HB, MO, KM, US-D, KT, MP, and AA: writing—review and editing. All authors contributed to the article and approved the submitted version.

## Funding

Funding for this research was provided by the Australian Government through the Youth Cancer Service. In addition to her clinical role at Sydney Youth Cancer Service, US-D is supported by an Early Career Fellowship from the Cancer Institute of New South Wales (ID: 2020/ECF1163) and an Early Career Fellowship from the National Health and Medical Research Council of Australia (APP1111800).

## Conflict of Interest

The authors declare that the research was conducted in the absence of any commercial or financial relationships that could be construed as a potential conflict of interest.

## Publisher’s Note

All claims expressed in this article are solely those of the authors and do not necessarily represent those of their affiliated organizations, or those of the publisher, the editors and the reviewers. Any product that may be evaluated in this article, or claim that may be made by its manufacturer, is not guaranteed or endorsed by the publisher.
