# Supplementary figures and images for: The Clinical Utility of the Adolescent and Young Adult Psycho-Oncology Screening Tool (AYA-POST): Perspectives of AYA Cancer Patients and Healthcare Professionals
Source: Front Psychol. 2022 May 6;13:872830. doi: 10.3389/fpsyg.2022.872830 (PMC9120841; doi:10.3389/fpsyg.2022.872830)

Supplementary Material


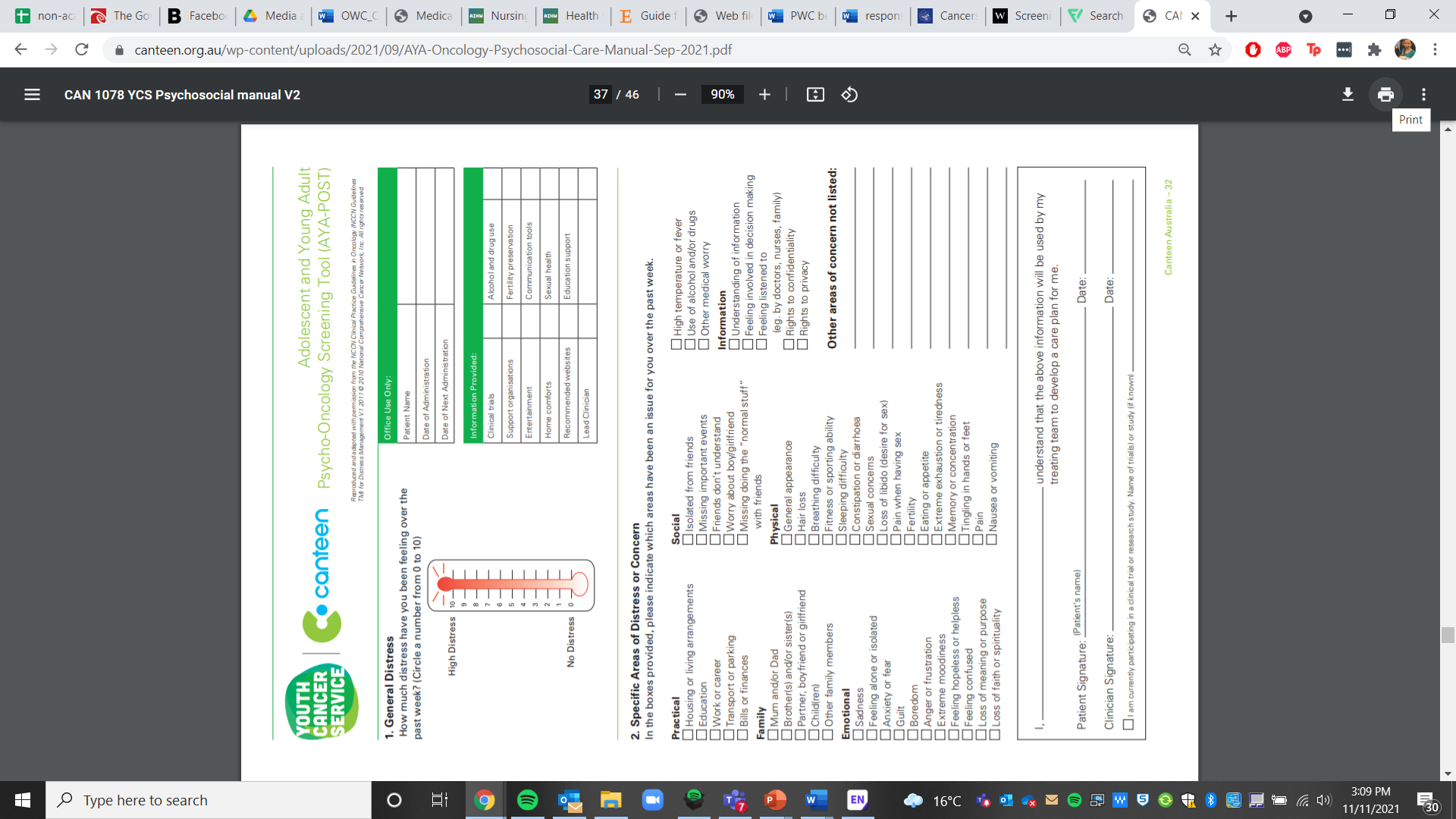

Supplement: Supplementary file 1 [file Table_1.DOCX]
